# Homing of endogenous bone marrow mesenchymal stem cells to rat infarcted myocardium via ultrasound-mediated recombinant SDF-1α adenovirus in microbubbles

**DOI:** 10.18632/oncotarget.23068

**Published:** 2017-12-08

**Authors:** Gaofeng Su, Liyun Liu, Lingjie Yang, Yuming Mu, Lina Guan

**Affiliations:** ^1^ Department of Echocardiography, First Affiliated Hospital of Xinjiang Medical University, Urumqi, China; ^2^ Xinjiang Key Laboratory of Medical animal Model Research, Clinical Medical Research Institute of First Affiliated Hospital of Xinjiang Medical University, Urumqi, China

**Keywords:** ultrasound targeted microbubble destruction (UTMD), gene transfection, acute myocardial infarction, SDF-1α, BMSCs

## Abstract

Stem cells can promote myocardial regeneration and accelerate the formation of new blood vessels. As such, transplanted stem cells represent a promising treatment modality for acute myocardial infarction (AMI). Stem cells spontaneously home to the infarcted myocardium using chemotaxis, in which the stromal cell-derived factor (SDF-1α) has been shown to be one of the most important chemokines. However, spontaneously secreted SDF-1α is short-lived, and therefore does not meet the needs of tissue repair. In this study, adenoviruses carrying *SDF-1α* genes were loaded on microbubble carriers and the adenoviruses were released into AMI rats by ultrasound targeted microbubble destruction. The possibility of *in vivo* self-transplantation of bone marrow mesenchymal stem cells (BMSCs) induced by overexpression of SDF-1α in the infarcted myocardium was explored by detecting the number of BMSCs homing from the peripheral blood to the myocardial infarcts. The concentration of SDF-1α in peripheral blood was significantly higher after transfection, and the number of BMSCs was significantly higher in the peripheral blood and infarcted area. Further analyses indicated that the number of homing BMSCs increased with increased SDF-1α expression. In conclusion, our results suggest that ultrasound mediated transduction of exogenous SDF-1α genes into myocardial infarcted AMI rats can effectively promote the homing of endogenous BMSCs into the heart. Moreover, the number of homing stem cells was controlled by the level of SDF-1α expression.

## INTRODUCTION

Stem cell transplantation in the treatment of acute myocardial infarction (AMI) has become a very promising treatment modality following the results of Hamano [1] and Strauer [2] where bone marrow stem cell transplantation was applied in the successful treatment of myocardial infarction. Transplanted bone marrow stem cells (BMSCs) reduce the area of myocardial infarction and promote ischemic cardiac function recovery by promoting myocardial cell regeneration and accelerating neovascularization [3–5]. However, in addition to the technical challenges of cell isolation and purification, exogenous transplantation of stem cells may lead to rejection by the immune system, pathogen transmission and other problems [6, 7]. More importantly, the homing of exogenous BMSCs does not efficiently reach the infarcted areas, which results in only limited improvement of cardiac function [8].

After myocardial infarction, stem cells can home spontaneously, via chemokine chemotaxis, to the damaged myocardium and become involved in physiological repair. Stromal cell-derived factor (SDF-1α) that arises from direct secretion by ischemic cardiomyocytes or paracrine secretion by BMSCs strongly promotes the homing of BMSCs to damaged sites [9, 10]. Moreover, SDF-1α also promotes neovascularization [11] and induces the differentiation of bone marrow stem cells into myocardiocytes [12]. It has also been demonstrated that a lack of SDF-1α molecules significantly reduces stem cell homing [13]. However, SDF-1α can be secreted spontaneously after a myocardial infarction event, but it can only be maintained for about 4 days. This is a naturally occurring but inefficient repair process in which cells home to myocardial tissue in need of regeneration [14]. Therefore, a key question remains of how to sustain the expression of SDF-1α in the damaged myocardium.

Considerable research into homing behavior of BMSCs has been performed in recent years. Some of these methods involve gene transfection to mobilize endogenous BMSCs [15], some involve using ultrasound targeted microbubble destruction (UTMD) to improve the transplantation efficiency of the exogenous BMSCs [16]. However, studies about promoting homing of endogenous BMSCs by combination UTMD and gene transfection are few in number. Actually, it has been found that dual-carriers prepared with microbubbles and virus carrying genes can effectively combine the advantages of microbubble carriers and the viral vectors. This dual-carrier approach not only achieves targeted gene transfection by UTMD, but also obtains satisfactory transfection efficiency [17, 18]. The biological effects generated by UTMD have proven to be a promising means of improving the homing of BMSCs to the ischemic myocardium [19].

In view of the limitations of exogenous stem cell transplantation and SDF-1α-induced BMSCs homing, we combined UTMD and gene transfection to foster continuous expression of the stem cell inducer SDF-1α in the infarct areas in order to stimulate more endogenous BMSCs to home to ischemic myocardium. This methodology represents a novel treatment of myocardial infarction and a novel application in the field of gene therapy. Building from previous studies [20], recombinant adenovirus carrying *SDF-1α* genes (pAd-*EGFP/SDF-1α*) was conjugated to microbubbles through an avidin-biotin bridge [21]. The conjugated gene carrier microbubble was used to evaluate the feasibility of UTMD in mediating *SDF-1α* gene homing into the heart of AMI rats and to further analyze the relationship between the number of homing BMSCs and SDF-1α expression.

## RESULTS

### DNA-binding capacity

**Figure 1:**
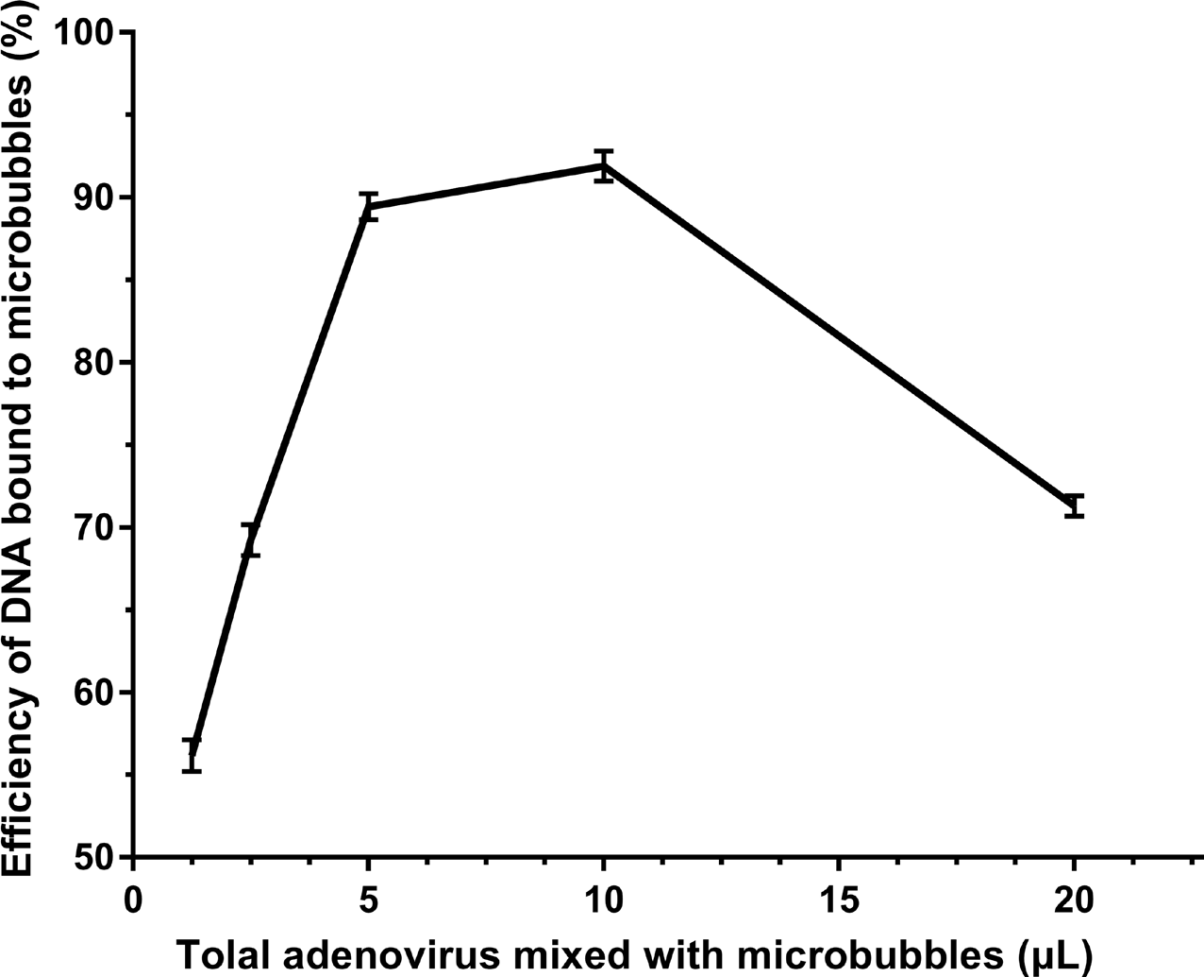
RT-PCR measurement of the efficiency of gene loaded onto microbubbles Various doses of adenovirus (1.25, 2.5, 5, 10, or 20 μl) carrying *SDF-1α* genes were added to 100 μl microbubbles. The titers of viruses was 1 × 10^9^ pfu/ml. The DNA-binding capacity of the microbubbles was quantified by comparing the amount of DNA bound to microbubbles and total gene added. (*n* = 6).

The amount of virus bound onto MBs was evaluated to measure the DNA loading capacity of MBs as described previously [22]. For this assay, RT-PCR analyses showed that as the dose of virus increased to 10 μL, the amount of virus bound to MBs also increased. There was no further increase in bound virus beyond 10 μl (Figure 1), which indicated that the DNA binding capacity of the MBs had reached saturation. Here, the maximum gene efficiency of MBs can reach 91%. Microbubble sizes were 2.8 ± 0.01 μm.

### Expression of SDF-1α in peripheral blood after gene transfection

**Figure 2:**
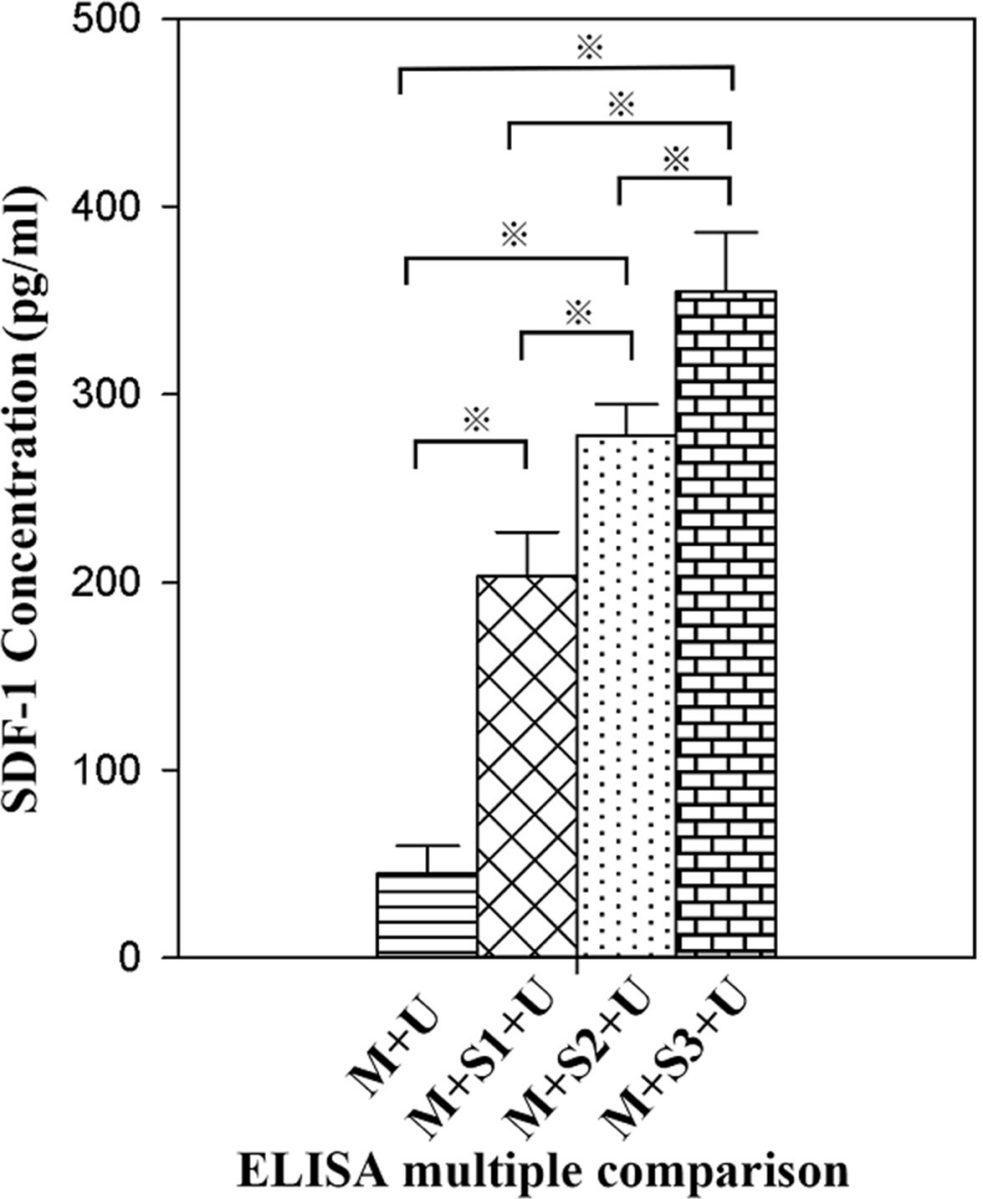
ELISA detection of peripheral blood SDF-1α concentrations All values are expressed as the mean ± SD. ^※^*P* < 0.01. (*n* = 10).

peripheral blood SDF-1α in the experimental group was higher than that of the control group, and the expression of SDF-1α increased with time after transfection. The SDF-1α concentration in the M + S3 + U group was 354.99 ± 31.38 pg/ml, which was 1.75 and 1.28 times that of the M + S1 + U and M + S2 + U groups, respectively. The differences between each two groups were statistically significant (*P* < 0.01). The results indicated that the expression of SDF-1α in AMI rat hearts was successfully induced by UTMD, and that the expression of SDF-1α increased with time after transfection (Figure 2).

### BMSCs concentrations in peripheral blood

**Figure 3:**
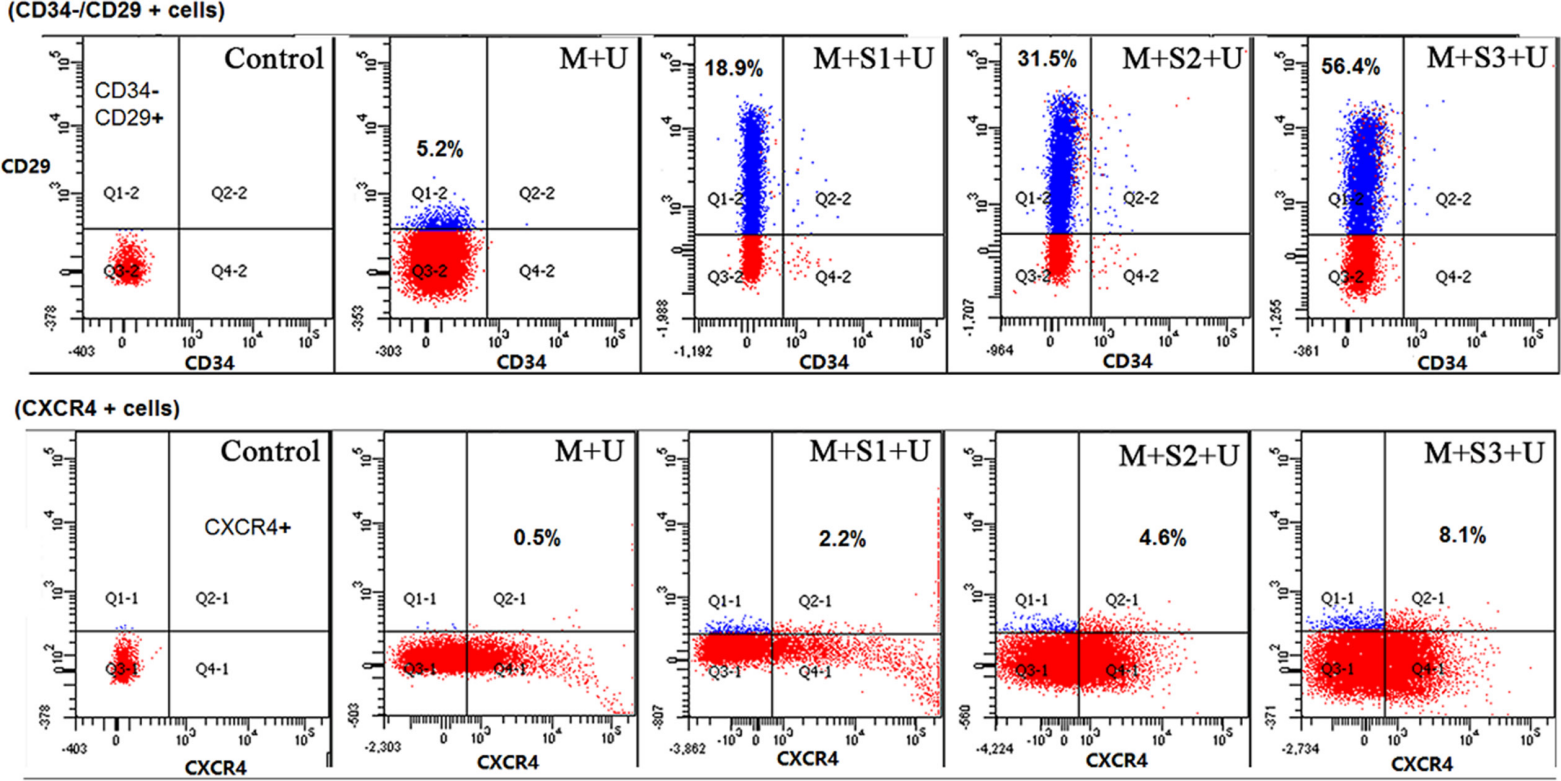
Flow cytometry analyses of BMSCs concentrations in peripheral blood CD34−/CD29 + and CXCR4 + are surface markers of BMSC and the graphs show the concentration of BMSCs in peripheral blood. CD29 was labeled with PE/Cy5, CD34 with PE and CXCR4 with Alexa Fluor^®^ 488. Specific values of CD34−/CD29 + cells and CXCR4 + cells were marked in the corresponding position of the squares. The concentration of BMSCs in peripheral blood significantly increased after transfection with *SDF-1α* genes. (*n* = 10).

The number of CD34−/CD29 + cells and CXCR4 + cells in peripheral blood significantly increased after transfection with *SDF-1α* genes, as determined using flow cytometry. With upregulation of the SDF-1α/CXCR4 pathway, more BMSCs migrated to the peripheral blood and the concentration of CD34−/CD29 + cells in M + S1 + U, M + S2 + U and M + S3 + U groups were significantly higher than those in the M + U group (3.65, 6.06, and 10.85 times of M+U, respectively). Further, concentrations of CXCR4 + cells of the M + S1 + U, M + S2 + U and M + S3 + U groups were 4.53, 9.39, and 16.57 times of M + U group, respectively. Differences between each two groups were statistically significant (*P* < 0.01) (Figure 3).

### The transfection of exogenous *SDF-1α* gene promoted BMSCs homing to the infarcted area

The expression of CD73, CD90, CD105 and CXCR4 in the infarcted myocardium was detected using BMSCs surface markers to evaluate the effects of exogenous SDF-1α overexpression on the homing of BMSCs to the infarcted area.

### Immunofluorescence

**Figure 4:**
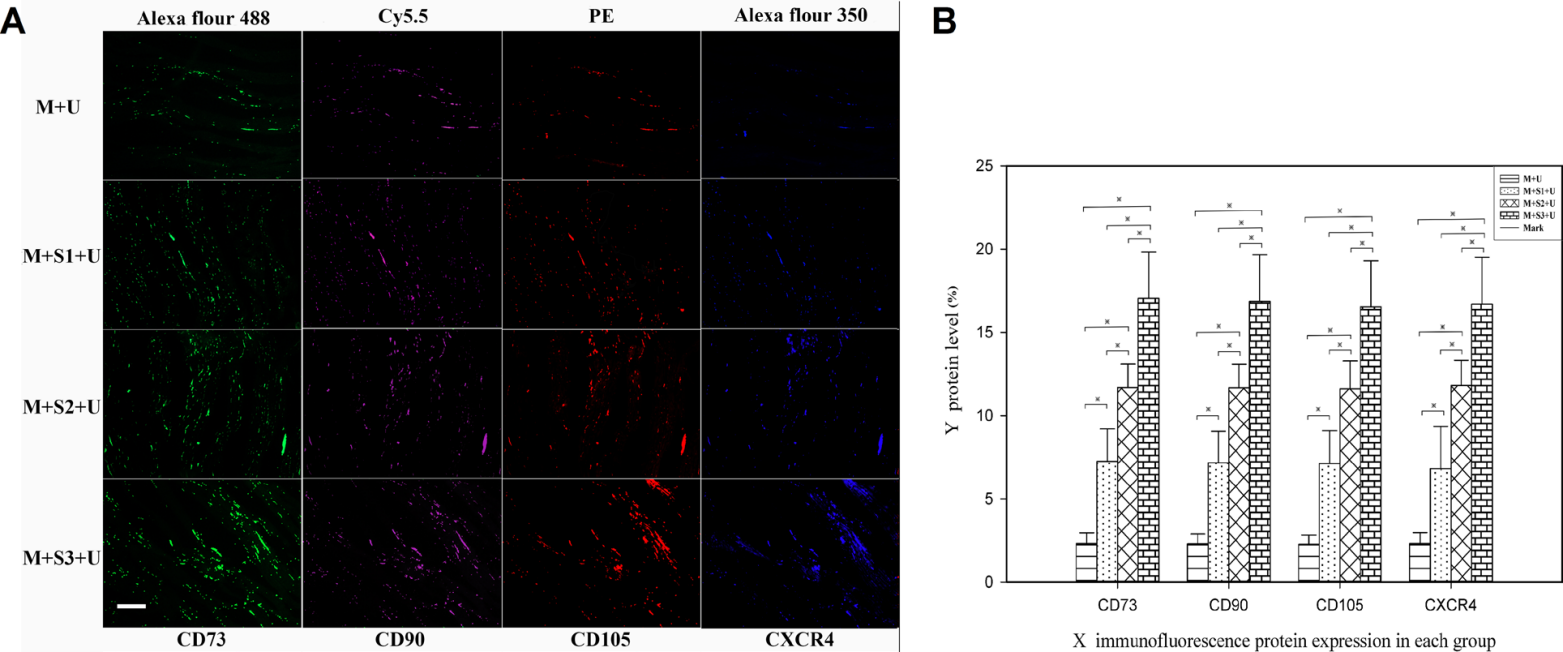
Immunofluorescent analysis of BMSC homing to myocardial infarcted areas (**A**) Immunofluorescent staining of BMSC surface proteins (CD73, CD90, CD105 and CXCR4) expressed in the infarcted areas. Scale bar = 200 μm. (**B**) The mean fluorescence area ratio of CD73, CD90, CD105 and CXCR4 represent the protein level after ultrasound mediated gene transfection. Graphs represent means ± SD. ^※^*P* < 0.05. (*n* = 10).

Immunofluorescence showed that CD73, CD90, CD105 and CXCR4 were all located on BMSCs cell surfaces, and the surface markers co-localized in the same microscopic fields of view (Figure 4). Semi-quantitative analysis using IPP6.0 software indicated that the fluorescent area of the experimental groups increased in comparison to the control group. Further, the expression of each biomarker increased gradually with time after transfection. The expression of CXCR4 proteins of the M + S1 + U, M + S2 + U and M + S3 + U groups were 2.93, 5.09, and 7.31 times that of the M + U group, respectively. The differences between each set of two compared groups were statistically significant (*P* < 0.05).

### RT-PCR

**Figure 5:**
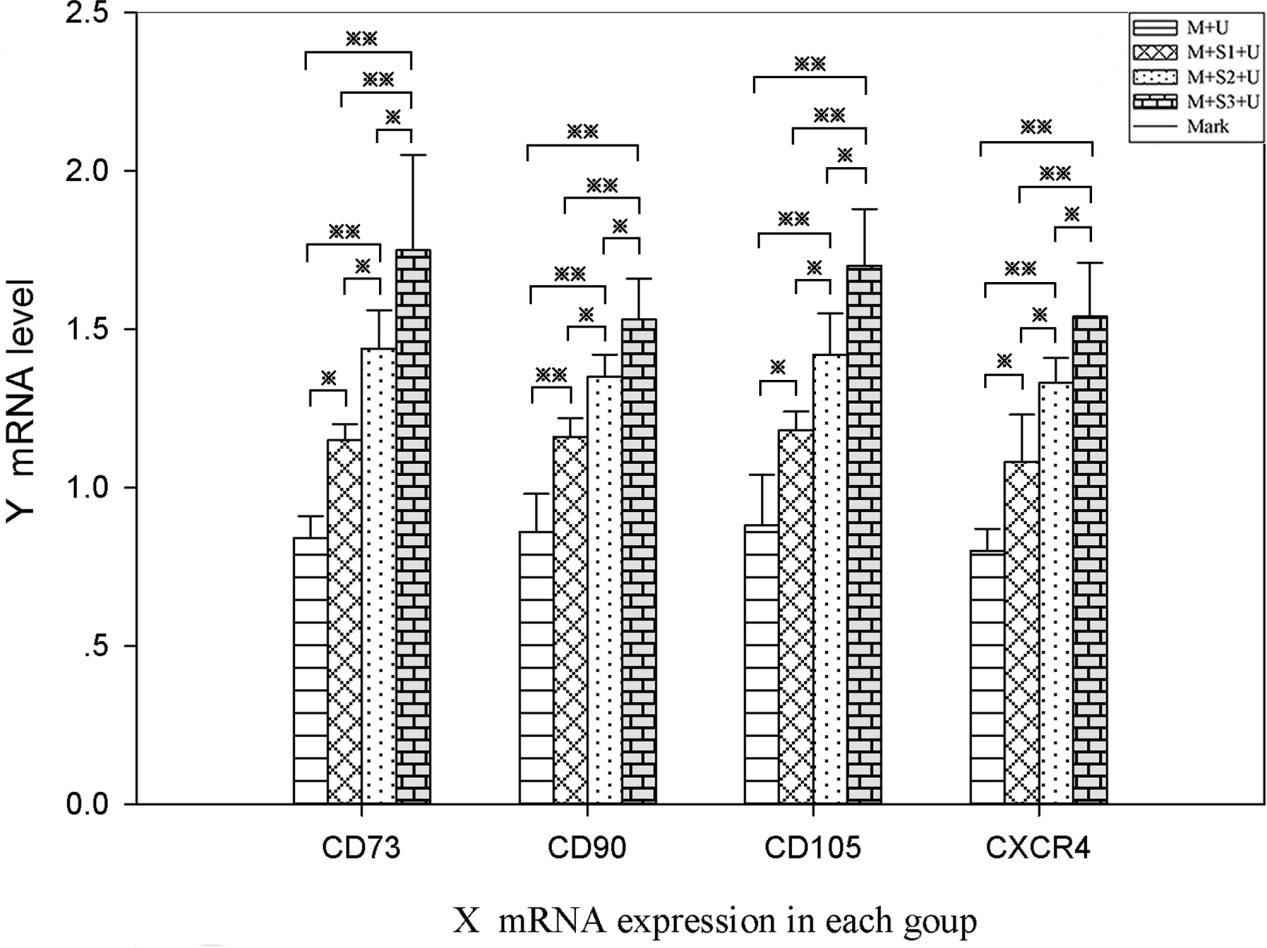
RT-PCR analysis of BMSC homing to myocardial infarcted areas mRNA expression levels of BMSC surface markers CD73, CD90, CD105 and CXCR4 as detected by RT-PCR. mRNA expression of CXCR4 in the M + S1 + U (1.08 ± 0.15), M + S2 + U (1.33 ± 0.08), and M + S3 + U (1.54 ± 0.17) groups were 1.35, 1.66 and 1.93 times that of the M + U (0.80 ± 0.07) group. ^※^*P* < 0.05, ^※※^*P* < 0.01. (*n* = 6).

mRNA expression of CD73, CD90, CD105 and CXCR4 genes in the infarct area was quantified using RT-PCR (Figure 5). The results indicated that exogenous *SDF-1α* gene overexpression could effectively promote the homing of BMSCs to the infarcted area. mRNA expression levels of CD73, CD90, CD105 and CXCR4 in the infarcted area significantly increased after transfection (*P* < 0.05), and the differences were statistically significant between the each two groups (*P* < 0.05). The results showed that the number of homing BMSCs increased with the increase in days after transfection. The expression levels of CD73, CD90, CD105 and CXCR4 on the surface of BMSCs in the same groups were similar, and the results were consistent.

### Western blot

**Figure 6:**
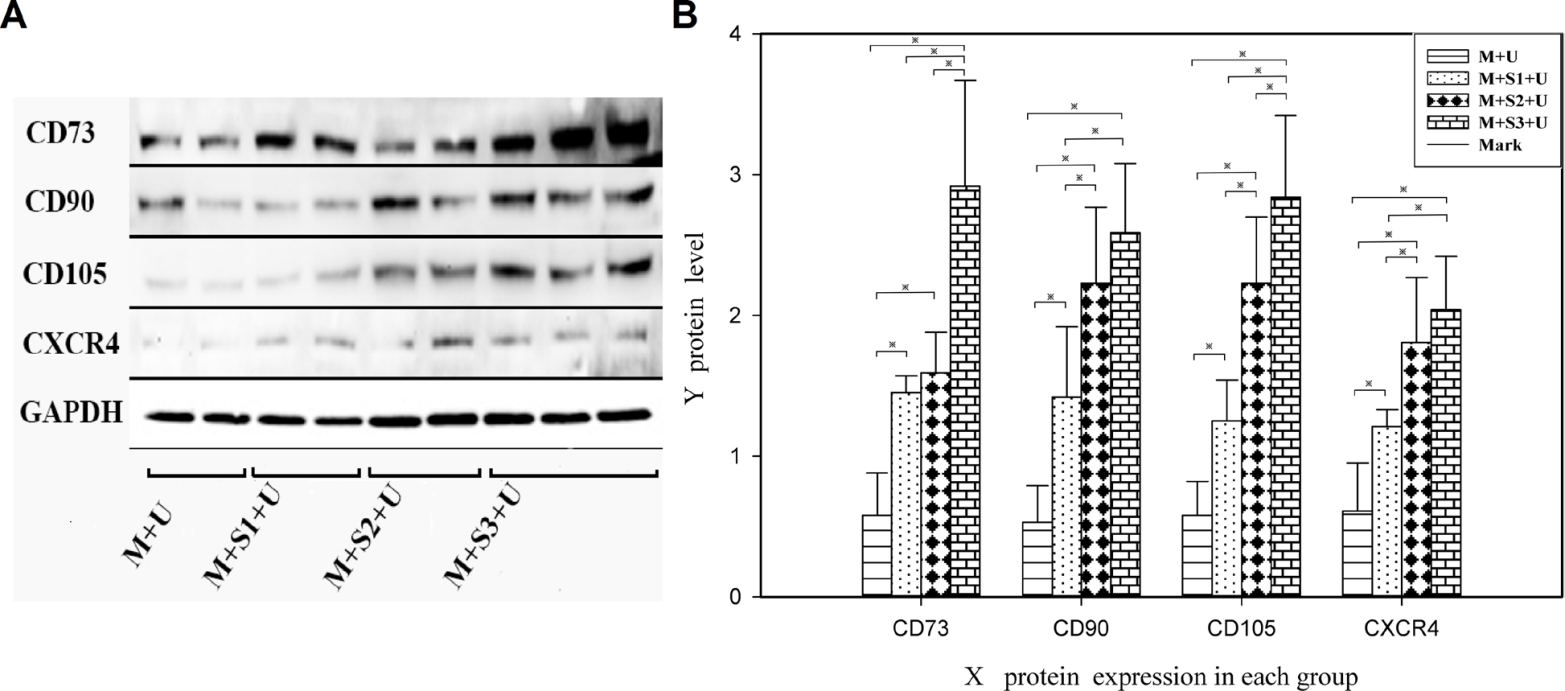
Western blot analysis of BMSC homing to myocardial infarcted areas (**A**) Western blot analysis of BMSC surface protein (CD73, CD90, CD105 and CXCR4) expression and (**B**) the signal intensities from these blot. Protein expression of CD73, CD90, CD105 and CXCR4 of the M + S1 + U, M + S2 + U and M + S3 + U groups increased in myocardial infarction areas compared to the M + U group. The differences between each two groups were statistically significant. ^※^*P* < 0.05. (*n* = 6).

The expression of CD73, CD90, CD105 and CXCR4 proteins in the infarcted zone increased in comparison to that of the control group (*P* < 0.05, Figure 6). The expression of CD105 proteins in the M + S3 + U group was 4.90, 2.27 and 1.27 times that of the M + U, M + S1 + U and M + S2 + U groups, respectively. The differences between each two groups were statistically significant (*P* < 0.01). Western blot detection of BMSCs surface marker proteins from infarcted myocardium showed similar results as the immunofluorescence analyses, indicating that protein expression of BMSCs surface markers CD73, CD90, CD105 and CXCR4 increased in the myocardial infarction area compared to the control group.

The expression levels of genes and proteins on BMSCs surfaces from the infarcted myocardium significantly increased after transfection compared to the control group. These results further suggested that the overexpression of exogenous *SDF-1α* genes significantly promoted the homing of BMSCs to the infarct area. It was also further demonstrated that the number of homing stem cells increased with increases in SDF-1α expression.

## DISCUSSION

Stem cell transplantation represents an appealing and effective means of treating myocardial infarction. However, previous methods of stem cell *in vitro* transplantation exhibit several limitations. In this study, recombinant adenovirus carrying *SDF-1α* genes were loaded onto microbubbles using the “avidin-biotin” crosslinking method. The microbubbles carrying the pAd-*EGFP/SDF-1α* gene were then targeted to the rat myocardium using UTMD technology. In contrast to previous methods of stem cell transplantation, this study utilized overexpression of transfected SDF-1α in order to promote the migration of BMSCs from bone marrow to infarcted areas and achieve self-transplantation. The results indicate that upregulation of the SDF-1/CXCR4 pathway by ultrasound-mediated dual-vector transfection of *SDF-1α* genes into the heart of AMI rats is feasible. Moreover, the results indicate that the methodology promotes the homing of BMSCs to the heart in a SDF-1α dose-dependent manner.

SDF-1α specifically anchors CXCR4 on the surface of the stem cell membrane, and the SDF-1α/CXCR4 pathway plays a key role in the migration of chemotactic BMSCs and homing to the inflammatory tissue area [23–25]. Until now, many methods have been found to upregulate SDF-1α expression, such as increasing the expression of hypoxia-inducible transcription factor-1(HIF-1) and ultrasound targeted microbubble destruction [26–28]. The above studies up-regulate SDF-1α by activating *SDF-1α* or inhibiting the degradation of SDF-1α protein indirectly, which all rely on the expression of the endogenous *SDF-1α* gene. Some other works up-regulated SDF-1α directly via plasmid gene delivery to the myocardium [28]. Taylor *et al.* [17] and Mueller *et al.* [18] also confirmed that reporter genes inserted into viruses and loaded on microbubbles showed much greater transfection efficiency after ultrasound irradiation. Importantly, the above methodologies reduce the immune risk posed by the virus [29]. In this way, ultrasound-mediated gene delivery (UGMD), especially dual-carriers prepared with microbubbles and gene-expressing viruses, merits further research [30]. However, studies about its ability to up-regulate the expression of SDF-1α have been relatively rare. In our study, after the injection of dual carrier microbubbles carrying *SDF-1α* in tail veins, the microbubbles were disrupted by ultrasound targeted irradiation which resulted in the release of the target gene in the target organ. The sonic effect during microbubble rupture [31] also opened pore channels and allowed viral entry into the cells, which further contributed to adenovirus infection, and consequently enhanced transfection efficiency. The results showed that the expression of SDF-1α and the number of homing BMSCs significantly increased in the experimental groups and that the number of homing BMSCs increased with increased SDF-1α expression. These results suggest that UTMD-mediated SDF-1α gene transduction into myocardial infarcted AMI rats can promote the homing of endogenous BMSCs into the heart. Moreover, repeating the operation enhanced the number of homing BMSCs further as the level of SDF-1α expression increased.

Some recent studies [16, 32] have demonstrated that UTMD could elevate secretion of some cytokines, including SDF-1α, while most of these studies aimed at short-term and immediate effects of UTMD. Li *et al.* [19] detected SDF-1α expression on day 7 after UTMD treatment, the results showed that a single UTMD operation has limited effect on secretion of SDF-1α: the SDF-1α expression of UTMD group increased by less than 2 fold control group levels. However, in our study, on day 7 after MI, we found the expression of SDF-1α had increased over 4 control levels, as had the homing of BMSCs, which suggested that gene transfection mediated by ultrasound might promote SDF-1α up-regulation and homing of BMSCs further. In addition, the avidin-biotin crosslinking method [33] used here achieved stable conjugation of microbubbles and adenovirus. Results of Nomikou *et al.* have shown that the binding efficiency acquired with this method is considerably higher than with electrostatic adsorption or other covalent binding methods [34]. The study provides a theoretical reference for UGMD-assisted BMSC homing.

Gene transfer and expression in target cells are theoretically a more fundamental and complete way of up-regulating of SDF-1α. There are, however, some limitations to this study. First, although this article addresses methods of promoting homing of BMSCs, an evaluation of the therapeutic effect would be appropriate. Second, due to the biological effects generated by UTMD, the conclusion could be more convincing if a myocardial infarction + UTMD group, in which the microbubbles were loaded with adenovirus without SDF-1α genes, were added. Third, we found that the level of SDF-1α expression may affect homing of BMSCs by increasing the number of days of transfection. However, further studies are still needed to understand the dose-effect relationship between SDF-1α and homing BMSCs. Finally, further analysis is needed to determine whether the virus dosage, which was determined using the maximum amount of gene-carrying microbubble detected in our previous experiments, coincides with the optimal dose needed for gene therapy. Additionally, in consideration of the ability of microbubbles to deliver genes to extravascular cells, preparation of nano-micro-bubbles will be our focus in future micro-bubble carrier studies. While the results reported here indicate a promising treatment methodology, SDF-1α-induced homing of BMSCs by gene transfection should be improved by further analyses of the conditions necessary for efficient application in a clinical setting.

In conclusion, microbubbles carrying genetically modified virus were successfully transfected using UTMD technology, and the expression of SDF-1α significantly promoted BMSC homing to the heart. A dose-effect association was confirmed between homing cells and the expression of SDF-1α, which provides new insights into the study of stem cell “self-transplantation” in the treatment of myocardial infarction.

## MATERIALS AND METHODS

### Experimental animals

Healthy 10-week-old Sprague-Dawley (SD) rats, that were male or female and weighed 250**–**300 g were used in the experiments and purchased from the Experimental Animal Research Center of the Xinjiang Medical University. The experimental protocol was approved by the Animal Ethics Committee of the First Affiliated Hospital of Xinjiang Medical University (approval number of IACUC-20141217009). Experiments were carried out in the Microsurgery Department of Experimental Animal Science of the First Affiliated Hospital of Xinjiang Medical University, which was accredited by the AAALAC international committee. The experimental study protocol follows the ARRIVE criteria [35]. Experiments were carried out in strict accordance with the Animal (Scientific Procedure) Law of the United Kingdom in 2013 and followed the “3R” principle of substitution, reduction and optimization to minimize damage to animals.

### Preparation of myocardial infarction animal models

Following the methodologies of Hou YL [36] and Kirihara Y [37] *et al.*, anesthesia was induced in rats via intraperitoneal injection of 2 mg/Kg atropine sulfate (Tianjin Jinyao Pharmaceutical Co., Ltd.). Dexmedetomidine (0.3 mg/kg body weight)/midazolam (4.0 mg/kg bw)/butorphanol (5.0 mg/kg bw) (Sigma) was then injected intraperitoneally ten minutes later. A 16G intravenous catheter (Introcan 16G, Braun Medical Co., Ltd., Germany) was used for endotracheal intubation of the animals and a rodent ventilator (HX-200, Chengdu Science and Technology Co., Ltd. Thailand Union) was used. After thoracotomy, the heart was exposed and the pericardium was stripped. Using the coronary vein as a landmark, the left atrial appendage was slightly elevated, and a 6/0 suture was inserted ∼2**–**3 mm from the start of the left coronary artery anterior descending branch with a depth of 0.5 mm. The suture came out near the pulmonary artery cones and ligation was made. Electrocardiograms showing typical ST-segment elevation, white left ventricular anterior wall color and hypokinesia were all used to assess ligation success. Intramuscular penicillin at 200,000 U was injected for 3 days after the surgery. Intraoperative and postoperative mortality was less than 10%.

### Experimental animal groups

40 SD rats were successfully modeled and randomly divided into control and experimental groups: myocardial infarction + ultrasound irradiation group (M + U/control group, *n* = 10); and the myocardial infarction + pAd-*EGFP/SDF-1α* gene microbubble + ultrasound irradiation group (M + S + U), which was further divided into three subgroups according to the number of days of transfection: 1 day of transfection (M + S1 + U, *n* = 10), 2 days of transfection (M + S2 + U, *n* = 10) and 3 days of transfection (M + S3 + U, *n* = 10).

### Ultrasound microbubble loading

The pAd-EGFP/SDF-1α was purchased from Nanjing Source Biotechnology Co., Ltd., Nanjing, China. The titers of viruses was 1 × 10^9^ pfu/ml and biotinylated by the company [38]. Targestar-SA microbubbles (Targestar TM-SA, Targeson, U.S.) were biotinylated and streptavidin coupled (Targeson) [39]. Biotinylated adenovirus was injected into microbubbles to prepare microbubble-virus complexes (pAd-EGFP/SDF-MBs). The amount of gene loaded onto microbubbles was evaluated as described previously [22]. For this assay, various doses of pAd-EGFP/SDF-1α (1.25, 2.5, 5, 10, or 20 μl) were added to 100 μl MBs and incubated for 20 min to enable adenovirus adsorption onto the MBs (pAd-EGFP/SDF-MBs). The mixture was allowed to stand for 10 min to extract 20 μL of the upper layer of the MB mixture and placed in a –20°C refrigerator as the unwashed sample for further testing. Then, the residual mixture was added to 2 ml PBS buffer and centrifuged at 1500 × g to form two phases: an upper, milky-white layer containing the MBs bound with adenovirus and a lower, clear layer containing unbound adenovirus. The lower layer was discarded and 20 μl of the upper layer was stored in a −20°C refrigerator as the washed sample. The microbubbles were removed by ultrasonic spallation (irradiation conditions: 1.5 MHz, 2 W, 15 s). All samples were diluted and the amounts of DNA in the washed samples were compared to that in the unwashed samples using real-time qPCR, to measure the amount of gene loaded onto MBs.

### Microbubble administration

The rats in the M + S1 + U, M + S2 + U, and M + S3 + U groups were injected with 100 µl of microbubble-virus mixture at 6 h (M + S1 + U), 6 h and 30 h (M + S2 + U), and 6 h, 30 h and 45 h (M + S3 + U) after infarction surgery. According to the manufacturer’s protocols (Targeson, USA), 100 µl is the maximum volume for rats. Microbubbles that were biotinylated and streptavidin coupled were incubated with biotinylated pAd-*EGFP/SDF-1α* after mixing at a 100:10 ratio. The optimal ratio of microbubbles to virus was 100:10, which achieved a 91% efficiency of gene loading as determined by RT-PCR assays. The conjugates were gently mixed at room temperature (18**–**25°C) for half an hour before administration.

### Ultrasound irradiation

As the infusion started, a GE Vivid 7 Dimension ultrasonic diagnostic apparatus and an i13l linear array probe (frequency 10**–**14 MHz) were used to detect the heart through the left chest wall according to previously described methods [40]. The instrument was set to a low mechanical index (MI = 0.4). When a large number of microbubble contrast agents filled the myocardium, the linear probe was immediately replaced with M3S probes (frequency: 2.0**–**3.5 MHz). The harmonic function was activated with an emission frequency of 1.3 MHz and a receiving frequency of 2.6 MHz. Additionally, the mode was changed to real-time myocardial contrast mode with adjustment of the mechanical index to 1.0. The FLASH (fastlow-angle shot) function was triggered every 3**–**6 cardiac cycles to break the microbubbles in the heart cavity and myocardial muscles. Each trigger was performed with an interval of 2**–**5 seconds to facilitate the next cardiac cycle with enough ultrasound contrast agent in the myocardial tissues. Ultrasound irradiation lasted 10 minutes [19]. All animals were sacrificed seven days after myocardial infarction, and the myocardial tissue of the infarcted area was immediately taken after intraperitoneal injection of 2.5% pentobarbital sodium solution.

### ELISA

The double antibody sandwich method was used to detect peripheral blood SDF-1α concentrations. The detection procedure was carried out according to the SDF-1α ELISA kit protocol (Shanghai Jining Industrial Co., Ltd.), and the concentration was calculated using a multiple function enzyme labeling system (Thermo Fisher Scientific, Inc).

### Flow cytometry

Flow cytometry (FACS Diva Version 6.1.3 US Becton Dickinson Company) was used to detect BMSC surface markers CD34−CD29 + and CXCR4 +, and the amount of BMSCs in peripheral blood. The PE/Cy5-labeled anti-mouse CD29 (Biolegend, CA, U.S.), PE-labeled mouse monoclonal CD34 (Abcam, U.K.), rabbit polyclonal CXCR4 primary antibody (Abcam), and goat polyclonal anti-rabbit Alexa Fluor^®^ 488 secondary antibody (Abcam) were used for flow cytometry analyses. The antibodies were added according to manufacturer’s instructions after collection of peripheral blood. Antibodies of the same species and isotype were used for experiments and those that were unrelated were used as negative controls. Cells were incubated for 30 min at 4°C in the dark, washed with cell staining buffer, and fixed in 2% paraformaldehyde at 4°C 30 min before flow cytometric identification of cell surface markers.

### RT-PCR

BMSCs resulting from homing were calculated based on BMSC surface markers in the infarct area. mRNA expression of CD73, CD90, CD105 and CXCR4 in the infarct area were detected using RT-PCR. Trizol reagent (Invitrogen, CA, U.S.) was used to extract total RNA from cardiac muscle tissues. cDNA was then transcribed from RNA and used as templates to amplify CD73, CD90, CD105 and CXCR4 genes. Specific RT-PCR steps were carried out according to manufacturer’s instructions of the TaKaRa reverse transcription kit using β-actin as a control. The amplification conditions were: 95°C 5 min, 35 cycles of 95°C 30 s, 54°C 45 s, and 72°C 1 min, and a final extension at 72°C for 5 min, followed by 4°C refrigerator cooling for 1 min. The PCR primer sequences are provided in Table 1. Agarose gels (2%) were used for electrophoresis and the results were analyzed in a Tanon gel analysis system.

**Table 1:** RT-PCR primer sequences

Gene	Forward primer (5′ −> 3′)	Reverse primer (5′ −> 3′)
CD73	GCTGCAGAGAACTTGATCCG	AAAACATGACTCTGGTGACCA
CD90	GCAGCCAGGAAGTGTTTTGA	AGGGCGACTACATGTGTGAA
CD105	CCTTTGGTGCCTTCCTCATT	CGATGCTGTGGTTGGTACTG
CXCR4	GCGAGCATTGCCATGGAAAT	GGAAGCAGGGTTCCTTGTTG
β-actin	CCCATCTATGAGGGTTACGC	TTTAATGTCACGCACGATTTC

### Western blot

Protein expression of CD73, CD90, CD105 and CXCR4 in the infarct area was detected using Western blot analysis. 100 mg of infarcted myocardium was added to 10 ml PBS for homogenization. The supernatant was then used to extract proteins and bovine serum albumin was used to plot a standard curve. Protein concentrations were determined via the Bicinchoninic acid (BCA) method. Proteins were separated by electrophoresis, transferred to a cellulose acetate membrane and blocked. The primary antibody (Abcam) was then added followed by the secondary antibody. After incubation, the membrane was washed, developed, scanned and analyzed using the NIH ImageJ software.

### Immunofluorescence

Immunofluorescence was used to detect CD73, CD90, CD105 and CXCR4 protein expression in the infarcted myocardium. The myocardial tissues were collected and embedded for frozen sections. After acetone fixation, the samples were incubated with 10% (volume fraction) goat serum for 30 minutes. The primary antibody (at 1:200 volume) was added, incubated overnight at 4°C and then washed three times with 0.1 M PBS for 5 min. The fluorescently labeled secondary antibody (at 1:200 volume) was added and incubated at 37**°**C for 60 min. The slides were then washed three times in PBS for 5 min each. The slides were sealed with glycerol buffer, observed using a fluorescence microscope and then photographed. IPP6.0 software was used to calculate the fluorescence area ratio.

### Statistical analysis

The SPSS 23.0 software was used for statistical analyses. All measurement data followed normal distribution and are expressed as the mean ± standard deviation (SD). Comparisons among multiple groups were tested using single factor analysis of variance and follow-up comparisons between two groups were conducted using the LSD method. Statistical significance was defined at *P* ≤ 0.05.
